# Laparoscopic Appendectomy for Acute Appendicitis With Adnexal Adhesion During the Third Trimester of Pregnancy: A Case Report

**DOI:** 10.7759/cureus.107997

**Published:** 2026-04-29

**Authors:** Kazunori Masahata, Takeya Hara, Rikuto Hirose, Yoshimasa Oyama, Kenshi Wasada

**Affiliations:** 1 Pediatric Surgery, Aizenbashi Hospital, Osaka, JPN; 2 Pediatric Surgery, Osaka University Graduate School of Medicine, Suita, JPN; 3 Obstetrics and Gynecology, Aizenbashi Hospital, Osaka, JPN; 4 Radiology, Aizenbashi Hospital, Osaka, JPN

**Keywords:** acute appendicitis, laparoscopic appendectomy, minimally invasive surgery, pregnancy, third trimester

## Abstract

Acute appendicitis during pregnancy is associated with maternal/fetal complications. Although laparoscopic appendectomy is widely accepted for managing appendicitis, limited data are available regarding laparoscopy in cases occurring during the third trimester of pregnancy. We present the case of a patient in the third trimester of pregnancy with acute appendicitis and adnexal adhesions who underwent laparoscopic appendectomy. The appendectomy was successful; however, dissection of the adhesions between the inflamed appendix and right adnexa led to prolonged operative and pneumoperitoneum times. The patient was discharged without complications, subsequently delivering a healthy infant at 38 weeks of gestation. This case shows that laparoscopic appendectomy can be performed safely during advanced pregnancy with careful consideration of lower intraperitoneal insufflation pressure, port arrangement, and surgical position. This report contributes to existing research regarding the safety of laparoscopic appendectomy in advanced pregnancy.

## Introduction

Acute appendicitis is the most common cause of acute abdomen during pregnancy, occurring in approximately one in 1000 pregnancies [1,2]. Perforated appendicitis during pregnancy is associated with maternal and fetal complications such as preterm delivery and fetal death [3]. Therefore, accurate and prompt diagnosis and appropriate surgical treatment are crucial. Appendectomy is the standard treatment for acute appendicitis during pregnancy [4-6]. Laparoscopic appendectomy is commonly used for the management of acute appendicitis in non-pregnant individuals. In the past, laparotomy was used during pregnancy owing to the limited visualization of the enlarged uterus and inflammatory adhesions.

However, laparoscopy for acute appendicitis during pregnancy has been gradually accepted due to advancements in surgeons’ technical skills and the benefits of small wounds, faster recovery, and shorter length of hospitalization. In recent years, laparoscopic appendectomy has been routinely performed in pregnant woman during the first and second trimesters of pregnancy, as recommended in the Society of American Gastrointestinal and Endoscopic Surgeons guidelines [4]. However, limited data are available regarding laparoscopy for appendicitis during the third trimester of pregnancy owing to the technical difficulties of performing laparoscopic appendectomy and the associated maternal and fetal risks.

Herein, we present the case of a pregnant patient with acute appendicitis and adnexal adhesions who underwent laparoscopic appendectomy during the third trimester of pregnancy.

## Case presentation

A 28-year-old pregnant woman (gravida 2, para 0) presented with right lower quadrant abdominal pain at 32 weeks of gestation. The onset of pain started the day before admission. The patient had not experienced any nausea and vomiting. Admission laboratory tests revealed an elevated white blood cell count (11,100/µL) and C-reactive protein (13.8 mg/dL) levels (Table 1).

**Table 1:** Laboratory data on admission day.

Parameter	Observed Value	Reference Range
White blood cell count	11,100 /µL	3,300–8,600 /µL
Hemoglobin	11.1 g/dL	11.6–14.8 g/dL
Hematocrit	34.7%	35.1–44.4%
Platelet count	218,000 /µL	158,000–348,000 /µL
Aspartate aminotransferase	11 U/L	13-30 U/L
Alanine aminotransferase	11 U/L	7-23 U/L
Lactate dehydrogenase	122 U/L	124-222 U/L
Blood urea nitrogen	7.4 mg/dL	8-20 mg/dL
Creatinine	0.46 mg/dL	0.46-0.79 mg/dL
Sodium	139 mEq/L	135–145 mEq/L
Potassium	4.2 mEq/L	3.6–4.8 mEq/L
Chloride	103 mEq/L	101–108 mEq/L
C-reactive protein	13.8 mg/dL	0–0.14 mg/dL

**Figure 1:**
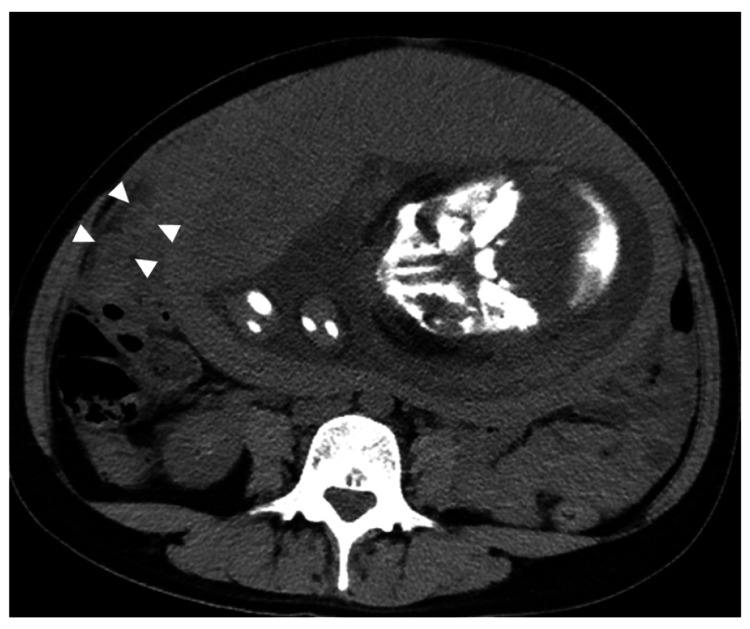
Abdominal computed tomography revealing the enlarged appendix at the right upper quadrant of the abdomen and spread of the inflammation to the surrounding areas (white arrowheads).

The fetal status was monitored using cardiotocography; no fetal distress was observed. Transabdominal ultrasonography was performed, but the appendix was not detected, resulting in an unclear diagnosis. Therefore, abdominal computed tomography was performed after explaining the risk of fatal radiation exposure to the patient. Computed tomography revealed an enlarged appendix in the right upper quadrant with visible spread of inflammation to the surrounding areas (Figure 1). A diagnosis of acute appendicitis was made, and a laparoscopic appendectomy was scheduled.

**Figure 2:**
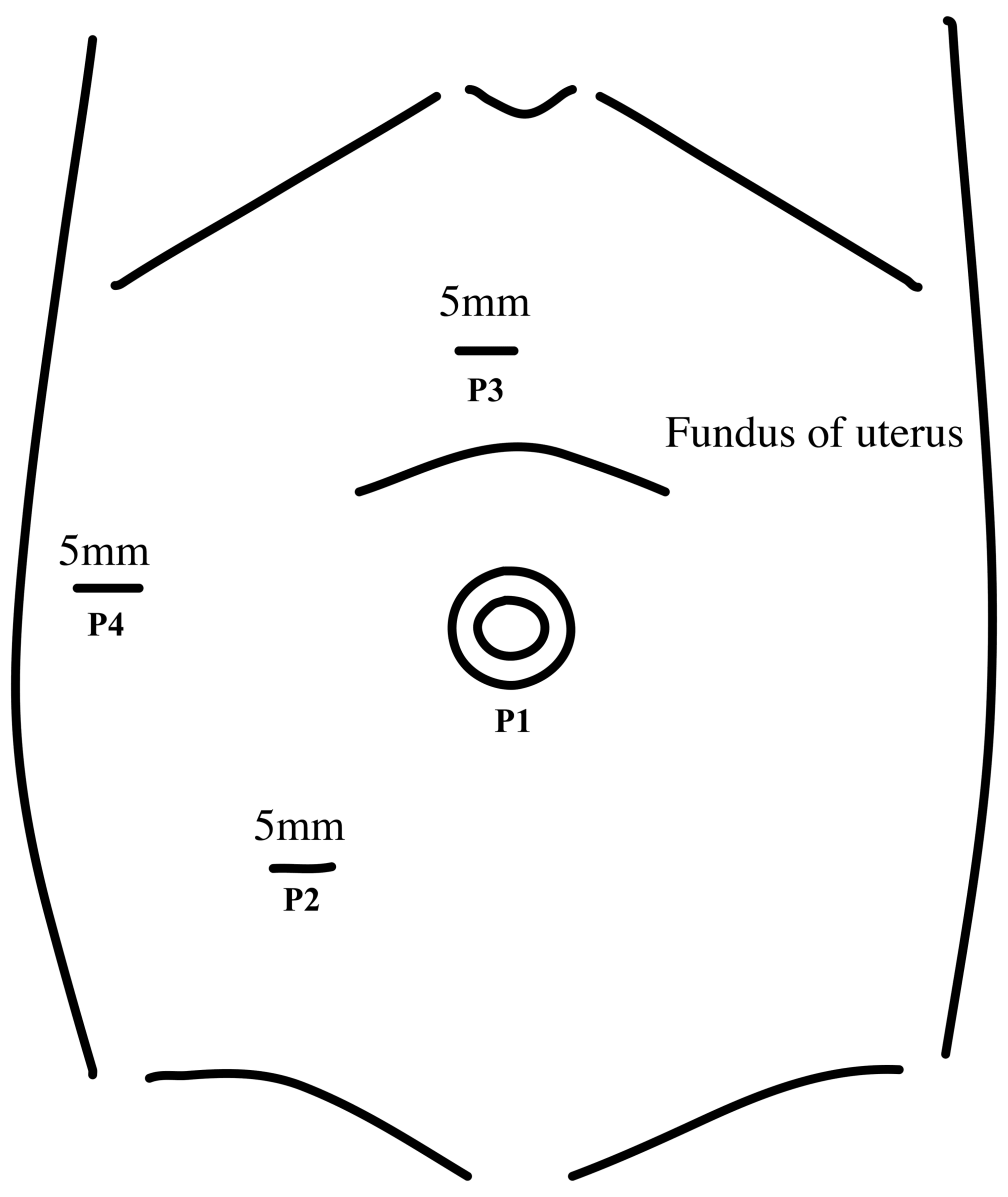
Port placement during laparoscopic appendectomy P1–P4 indicate the port sites. A multiple-instrument single-access port (Applied Medical Resources Corporation, Rancho Santa Margarita, California, United States) was first inserted into the peritoneal cavity at the umbilicus (P1). Subsequently, two working ports were placed under direct visualization of the scope at the level of the right lower quadrant of the abdomen (P2) and the epigastric site (P3). An additional port was placed in the right upper quadrant of the abdomen (P4).

Under general anesthesia, the patient was placed in the left semi-lateral position to avoid compression of the inferior vena cava by the gravid uterus. A 3-cm vertical skin incision was made at the umbilicus, followed by insertion of a multiple-instrument single-access port (Applied Medical Resources Corporation, Rancho Santa Margarita, California, United States) into the peritoneal cavity. Subsequently, 12- and 5-mm assistant ports were placed in the gel port (Figure 2).

**Figure 3:**
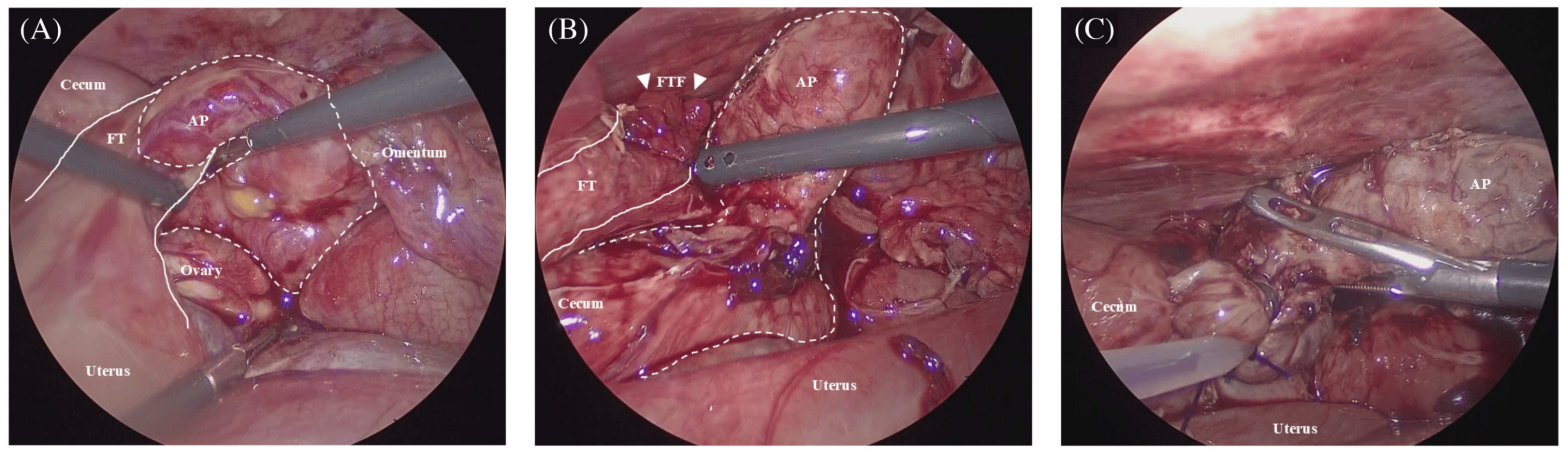
Intraoperative findings (A) The inflamed appendix (dashed line) has wrapped around the right adnexa (solid line) and adhered to the surrounding tissue. (B) Laparoscopic dissection of the adhesions between the appendix (dashed line) and the right adnexa including the fallopian tube (solid line) and fimbria (arrowheads), was successfully performed. (C) The appendix was excised at its base using endoloops. AP, appendix; FT, fallopian tube; FTF, fallopian tube fimbria

Carbon dioxide insufflation was set to 10 mmHg. The laparoscope was inserted into the abdomen via the umbilical single-access port. Purulent intraperitoneal fluid was aspirated from the right lower quadrant of the intra-abdominal cavity. Adhesions were noted and carefully dissected from the abdominal wall via the umbilical single-access port. Carbon dioxide insufflation was increased to 12 mmHg to account for the intra-abdominal space being occupied by uterine enlargement and inflammatory adhesions, impacting surgical field visualization. Under direct scope visualization, two working ports were placed at the level of the right lower abdominal quadrant and epigastric site (Figure 2). The appendix was severely inflamed and adhered to the right adnexa, making dissection difficult (Figure 3A).

Therefore, an additional port was placed in the right upper quadrant. Laparoscopic dissection revealed that the appendix was wrapped around the fallopian tube and fimbriae (Figure 3B). The appendix was excised at its base using endoloops (Figure 3C). Laparoscopic peritoneal lavage was completed, and a drainage tube was placed. The total operative time was 175 minutes; the pneumoperitoneum time was 132 min at 10-12 mmHg. No intra- or postoperative complications were observed. The patient was discharged on postoperative day 10 and ultimately delivered a healthy infant at 38 weeks of gestation via cesarean section for breech presentation. The absence of new intra-abdominal adhesions was confirmed during the cesarean. 

## Discussion

We successfully performed laparoscopic appendectomy in a pregnant patient with acute appendicitis and adnexal adhesions during advanced pregnancy. Owing to the use of lower intraperitoneal insufflation pressure, a specific port arrangement, and the left semi-lateral operative position, laparoscopic appendectomy was feasible and safely performed without conversion to laparotomy or maternal and/or fetal complications. However, the laparoscopic approach should be considered carefully when managing patients with appendicitis during advanced pregnancy.

When performing laparoscopic surgery in pregnant patients with an elevated uterine fundus above the umbilical level, the initial port insertion has a high risk to iatrogenically injury the enlarged uterus or other organs. The most important consideration during the placement of the first port is the insertion procedure. We recommend using the open (Hasson) method to insert the port at the umbilicus. Unlike the Veress needle technique and direct trocar insertion, the Hasson method allows direct visualization of the uterus and has lower risk of uterine injury. In addition, we used a multiple-instrument single-access port which we first inserted into the peritoneal cavity. This port has been widely used for single-site laparoscopic surgery, as only small incisions are required (1.5-4 cm) [7]. The Alexis wound protector/retractor of the port offers atraumatic retraction and a flexible fulcrum, allowing easy movement of surgical instruments during laparoscopy.

To minimize interference with the uterus, the port sites must be altered from standard positions in accordance with the enlarged uterus during advanced pregnancy [6]. Although port insertion on the left side remains feasible in early pregnancy, centering the port placement on the right side of the abdomen is crucial in advanced pregnancy. In the present case, we inserted four ports in specific positions, allowing the formation of a coaxial/triangular formation. This approach provides optical surgical exposure, specifically in advanced pregnancy wherein clinicians must consider uterine enlargement and appendiceal displacement. We suggest that insertion of additional ports should be considered in laparoscopy in pregnant patients with appendicitis and concomitant severe inflammatory adhesions. The Society of American Gastrointestinal and Endoscopic Surgeons guidelines recommend limiting insufflation pressure to 10-15 mmHg during laparoscopic appendectomy. This decreases the risks of fetal acidosis caused by reduced uteroplacental blood flow. Moreover, limiting insufflation pressure in pregnant patients decreases the risk of damage to the pulmonary physiology [4]. At our institute, laparoscopic appendectomy during pregnancy is performed using 10-12 mmHg, as the intra-abdominal pressure under such conditions is adequate. Additionally, the patient is positioned in the left semi-lateral position to reduce compression of the vena cava and aorta [8].

In this case, laparoscopic dissection between the appendix and adnexa required prolonged operative and pneumoperitoneal times. Prolonged procedural times compress the uterus, increasing the risk of adverse outcomes for the mother and fetus. Although ipsilateral adnexectomy was discussed and could theoretically have been performed in the present case, this approach was not considered feasible owing to the importance of preserving fertility. Conversion to an exploratory laparotomy was considered high risk, as this would have required a large incision. Upon consultation with the obstetricians, we continued the laparoscopic approach without adnexectomy or conversion to laparotomy and completed the procedure without incident. In hindsight, given the increased risk of adverse outcomes, we should have converted the procedure to laparotomy or right adnexectomy. Our experience with this patient suggests that surgeons should focus on efforts to reduce operative and pneumoperitoneum times in similar patients in advanced pregnancy. 

## Conclusions

This case shows that laparoscopic appendectomy can be performed safely during advanced pregnancy with careful consideration of lower intraperitoneal insufflation pressure, port arrangement, and surgical position. This report provides a foundation for expanding the use of laparoscopic surgery in managing appendicitis during advanced pregnancy by detailing the challenges faced during this patient encounter.
